# What Should We Be Recommending for the Treatment of Enteric Fever?

**DOI:** 10.1093/ofid/ofad179

**Published:** 2023-06-02

**Authors:** Christopher M Parry, Farah N Qamar, Samita Rijal, Naina McCann, Stephen Baker, Buddha Basnyat

**Affiliations:** Clinical Sciences, Liverpool School of Tropical Medicine, Liverpool, United Kingdom; Medical Microbiology, Alder Hey Children’s Hospital, Liverpool, United Kingdom; Centre for Tropical Medicine and Global Health, Nuffield Department of Medicine, University of Oxford, Oxford, United Kingdom; Department of Paediatrics and Child Health, Aga Khan University, Karachi, Pakistan; Oxford University Clinical Research Unit-Nepal, Kathmandu, Nepal; Hospital for Tropical Diseases, UCL, London, United Kingdom; Oxford Vaccine Group, Department of Paediatrics, University of Oxford, Oxford, United Kingdom; Cambridge Institute of Therapeutic Immunology and Infectious Disease, Department of Medicine, University of Cambridge, Cambridge, United Kingdom; Centre for Tropical Medicine and Global Health, Nuffield Department of Medicine, University of Oxford, Oxford, United Kingdom; Oxford University Clinical Research Unit-Nepal, Kathmandu, Nepal

**Keywords:** antimicrobial resistance, enteric fever, RCTs, systematic reviews, XDR typhoid

## Abstract

Patients with suspected enteric (typhoid and paratyphoid) fever are predominantly managed as outpatients in endemic regions. Nonspecific clinical presentation, lack of accurate diagnostic tools, and widespread antimicrobial resistance makes management challenging. Resistance has been described for all antimicrobials including chloramphenicol, amoxycillin, trimethoprim-sulfamethoxazole, ciprofloxacin, ceftriaxone, and azithromycin. No significant differences have been demonstrated between these antimicrobials in their ability to treat enteric fever in systematic reviews of randomized controlled trials (RCTs). Antimicrobial choice should be guided by local resistance patterns and national guidance. Extensively drug-resistant typhoid isolates require treatment with azithromycin and/or meropenem. Combining antimicrobials that target intracellular and extracellular typhoid bacteria is a strategy being explored in the Azithromycin and Cefixime in Typhoid Fever (ACT-SA) RCT, in progress in South Asia. Alternative antimicrobials, such as the oral carbapenem, tebipenem, need clinical evaluation. There is a paucity of evidence to guide the antimicrobial management of chronic fecal carriers.

## Teaser

Management of patients with enteric (typhoid and paratyphoid) fever is challenging. Antimicrobial choice is guided by local resistance patterns. Future regimens may require antimicrobial combinations or new agents. There is limited evidence to guide the antimicrobial management of chronic carriage.

Enteric (typhoid and paratyphoid) fever is a significant cause of febrile illness in regions of the world where the population lack access to clean water and adequate sanitation [1]. It is also a sporadic problem in those returning from travel to these areas. Enteric fever affects more than 14 million people globally each year, predominantly children and young adults, including an estimated 7 million living in South Asia [2]. Antimicrobials transform this prolonged febrile illness, with a mortality that varied between 10% and 30%, to a treatable syndrome in which symptoms resolve within 1 week and a mortality of <1% [1]. This paradigm is challenged by the relentless emergence of resistance to all currently used antimicrobials [3].

## WHAT IS THE DISEASE AND HOW IS IT DIAGNOSED?

Enteric fever is caused by infection with the human restricted bacteria *Salmonella enterica* serovar Typhi and *S. enterica* serovar Paratyphi A with transmission arising from human to human by the fecal-oral route [1]. Symptoms typically start 1 to 2 weeks after exposure (range, 3–60 days). The incubation period for paratyphoid fever is shorter than typhoid but the disease symptoms are comparable. A fever of gradual onset reaching 39°C–40°C after 5–7 days is typical. Associated symptoms may include diarrhea, nausea, vomiting, and poorly localized abdominal pain, headache, cough, and malaise [4]. Most patients are treated with antimicrobials as an outpatient at this stage and will recover. In the smaller number of patients who do not get effective treatment, the illness continues after the first week, with persistent fever, weakness, weight loss, and a clouded mental state occurs. Complications may develop such as gastrointestinal bleeding, nephritis, hepatitis, intestinal perforation, and encephalopathy and hospitalization is required [4]. In recent systematic reviews of enteric fever reports, the pooled prevalence of complications estimated among patients hospitalized with typhoid fever was 27% (95% confidence interval [CI], 21%–32%) [5], and the mean overall case fatality was 2.49% (95% CI, 1.65%–3.75%) and 4.45% (95% CI, 2.85%–6.88%) in patients who were hospitalized [6]. The decision to refer to hospital and use intravenous rather than oral treatment depends on a clinical assessment of the patient. Indications might include whether a patient is vomiting and unable to take oral medication, is clinically unstable, or has developed complications or instances in which the diagnosis is uncertain [4].

Other infections, such as malaria, influenza, COVID-19, dengue, chikungunya, scrub and murine typhus, brucellosis, and leptospirosis, may cause a similar initial illness. Distinguishing enteric fever from these other infections is difficult due to the nonspecific clinical presentation [7]. Although a blood culture is the optimum method to confirm a diagnosis of enteric fever, in a systematic review of published studies, blood culture has a reported sensitivity of 61% (95% CI, 52%–70%) [8]. The reality in endemic areas is that the sensitivity of blood cultures is less than this value, and suitable microbiology facilities for conducting such tests are often unavailable. Point-of-care serological rapid tests are available but lack diagnostic accuracy [9]. Early antimicrobial treatment to cover likely causes is therefore given empirically as an outpatient in patients with fever for 3 to 4 days and suggestive symptoms. This inevitably leads to antimicrobial overuse and potentially incorrect treatment.

## WHAT ANTIMICROBIALS CAN BE USED TO TREAT ENTERIC FEVER?

Most patients with enteric fever can be treated with an oral antimicrobial as an outpatient if treatment is started in the first week of illness. Only approximately 1 in 10 patients require hospital admission and parenteral treatment when danger signs develop, such as persistent vomiting, altered consciousness, or other complications [1]. Oral chloramphenicol, ampicillin/amoxycillin, and trimethoprim-sulfamethoxazole were commonly used and found to be effective before the 1990s. Multidrug resistance (MDR), with plasmid-mediated resistance to all these 3 options, appeared in the late 1980s and has spread to many countries [1, 3]. The fluoroquinolone (FQ) class of antimicrobials, which includes ciprofloxacin, in turn became a common choice to treat enteric fever, but low-level resistance (indicated by resistance to nalidixic acid or pefloxacin) and high-level resistance has become widespread in South Asia and some areas of sub-Saharan Africa such that they are no longer a reliable choice [1, 3]. Parenteral ceftriaxone and oral cefixime are effective and particularly used in children. Ceftriaxone has been considered a reliable option when resistance to other drugs is uncertain. Azithromycin is a further oral drug that has become commonly used drug for treating enteric fever over the last 20 years. Sporadic reports of resistance to azithromycin have been reported [10, 11].

Since 2016, confidence in ceftriaxone as a reliable option has been dented by outbreak of extensively drug-resistant (XDR) typhoid strain in Pakistan [12]. These organisms are resistant to chloramphenicol, ampicillin/amoxycillin, trimethoprim-sulfamethoxazole, ciprofloxacin, and ceftriaxone/cefixime. The cephalosporin resistance is mediated by carriage of a plasmid-mediated *bla_CTX-M-15_* extended-spectrum beta-lactamase (ESBL) gene [13]. These isolates have remained susceptible to oral azithromycin and parenteral meropenem. Infections with XDR typhoid have been reported in other countries and are usually associated with returning travelers [14]. Sporadic isolates with resistance to ceftriaxone have also been reported from locations outside Pakistan [15, 16], and there have also been reports of *S.* Typhi isolates carrying the carbapenem resistance genes *VIM* and *GES* [17].

Resistance has been reported to all the commonly used antimicrobials for treating enteric fever. The international spread of resistance has been dominated by the H58 genomic haplotype of *S.* Typhi although other genotypes have also been implicated [18]. Resistance is associated with treatment failure, an increased risk of complications, and increased potential for transmission due to prolonged fecal carriage [1, 19, 20].

Patterns of resistance vary by location and over time [3]. Treatment choices should take account of local resistance patterns, if known, and national guidelines where available [21]. In some areas, resistance to previously used antimicrobials, such as chloramphenicol and trimethoprim-sulfamethoxazole, has declined, and there have been calls to use these antimicrobials again [1].

## IS THERE EVIDENCE THAT ONE ANTIMICROBIAL IS BETTER THAN ANOTHER?

Three published Cochrane Systematic Reviews have studied antimicrobial efficacy in enteric fever from the perspective of the FQ (such as ciprofloxacin, ofloxacin, and gatifloxacin), azithromycin, and cephalosporins (such as ceftriaxone and cefixime) [22–24]. In these reviews, the authors found limited evidence to make firm conclusions over the advantage of one antimicrobial over another. The reviews all comment that many of the randomized controlled trials (RCTs) were conducted >20 years ago and some are of poor quality. Most trials were not double-blinded, and many recruited small numbers of trial participants for each comparison, leading to wide confidence intervals for measured outcomes that make it impossible to make firm conclusions on the presence or absence of important differences. All studies have relied on blood culture to confirm the diagnosis, which lacks sensitivity and is not a perfect reference standard. Most trials included only inpatients, which does not reflect the outpatient setting where enteric fever cases are usually treated. The changing pattern of resistance over time, the paucity of data on post-treatment fecal shedding, the lack of RCTs in severe enteric fever, and the lack of agreed core outcome indicators are further limitations.

A systematic review of RCTs that included a FQ in at least 1 of the arms identified 26 RCTs with 3033 participants [22]. The major fluoroquinolones studied included ciprofloxacin, ofloxacin, and gatifloxacin; all were found to be effective in treating enteric fever. A 7-day course of any FQ appeared to be at least as effective as a 14-day course of chloramphenicol with respect to clinical and microbiological clinical failures (8 trials, 916 participants). Trials comparing trimethoprim-sulfamethoxazole or amoxycillin with FQ were small and lacked adequate reporting of levels of resistance. Comparisons of 5–7 days of an FQ with 3 days of ceftriaxone were also too small to demonstrate important differences if they exist. Of note, the FQ gatifloxacin was effective in treating enteric fever in areas with high levels of low-level FQ resistance. However, concerns about adverse events and the emergence of further resistance have meant this FQ is no longer used.

The systematic review of trials that included azithromycin in at least 1 of the arms identified 7 trials with 773 participants [23]. Overall, the authors concluded that azithromycin appeared to be as good as the other comparator drugs, including chloramphenicol, ceftriaxone, and FQ, for most outcomes. An analysis of 4 RCTs with 564 participants, when azithromycin was compared with an FQ (ciprofloxacin, ofloxacin, and gatifloxacin), favored azithromycin for clinical failures (odds ratio, 0.48; 95% CI, .26–.89]), but there was no statistical difference for microbiological failure, relapse, and duration of fever. These RCTs included participants infected with MDR *S.* Typhi isolates and with low level-resistance to FQ.

A recent systematic review of the use of cephalosporins in enteric fever identified 27 trials with 2231 participants [24]. Ceftriaxone was an effective and well tolerated treatment for enteric fever with an efficacy that was similar to chloramphenicol and FQs. Ceftriaxone has been compared with azithromycin in 3 RCTs involving 196 children. No significant difference in the relative risk (RR) of clinical failure (RR, 0.40; 95% CI, .10–1.59) or microbiological failure (RR, 1.98; 95% CI, .35–11.22) was detected. Relapse at 30 days was found to be significantly more likely in the ceftriaxone arm (RR, 11.9; 95% CI, 2.17–65.06). The evidence for cefixime in treating enteric fever is mixed and it may not perform as well as FQs.

The WHO Essential Medicines Expert Committee reviewed the evidence for the comparative efficacy of different antimicrobials in enteric fever in 2019 and concluded that ciprofloxacin, ceftriaxone, and azithromycin should be considered first-choice treatments for enteric fever on the core list of the Essential Medicines List (EML) and EML for children [25]. Ofloxacin was not recommended because it demonstrated similar performance to ciprofloxacin, and the evidence was not considered strong enough to recommend cefixime. The Expert Committee also recommended that knowledge of the local resistance patterns for *S.* Typhi and *Salmonella* Paratyphi was critical for making empiric treatment choices in the treatment of enteric fever. Ciprofloxacin is only recommended as a first choice in settings with a low prevalence of FQ resistance. A low prevalence was not defined in the document but is often considered as less than 10%.

## HOW SHOULD WE MANAGE EXTENSIVELY DRUG-RESISTANT TYPHOID?

The appearance of XDR typhoid in Pakistan in 2016 created a considerable challenge for local clinicians [12]. A retrospective review of 81 patients with culture-confirmed XDR typhoid admitted at the Aga Khan University hospitals was reported in 2020 [26]. Most patients (n = 45; 56%) were male and the mean age of the cases was 8.03 years (with a range of 1–40). A total of 66 of 81 patients were treated as an inpatient. Fever and vomiting were the most common symptoms at the time of presentation. The mean time to defervescence was 7.1 (95% CI, 5.5–8.6) days in 22 patients treated with oral azithromycin with 1 treatment failure, 6.7 (95% CI, 4.7–8.7) days in 20 patients treated with intravenous meropenem alone with no treatment failures, and 6.7 (95% CI, 5.5–7.9) days for the 39 patients treated with a combination of azithromycin and meropenem and 3 treatment failures. The authors did not discern any important differences between each regimen. It is notable that the average cost of treatment per day for azithromycin was US $5.87, considerably less than the US $88.46 daily cost of meropenem.

Because enteric fever is mostly treated with oral antimicrobials in outpatients, the presence of susceptibility to only 1 oral antimicrobial—azithromycin—has been a particular concern. The carbapenems need to be given intravenously and are expensive. Giving intravenous meropenem treatment to an outpatient would not be possible. Meropenem, with or without azithromycin, has now been widely used for treating XDR enteric fever, and some imported infection case reports describe patients who have not improved on meropenem alone, but they have improved when a second agent is added [14, 21]. The use of meropenem for XDR enteric fever has yet to be assessed in an RCT.

## SHOULD WE BE USING MORE THAN ONE ANTIMICROBIAL TO TREAT ENTERIC FEVER?

Studies in Vietnamese patients with typhoid fever have indicated that *S.* Typhi infection is a mixture of an intracellular and an extracellular infection. In 365 patients with blood culture-confirmed typhoid fever, the median number of *S.* Typhi bacteria in blood was 1 colony-forming unit (CFU)/mL (range, <0.3 to 387 CFU/mL). A mean of 63% (95% CI, 58%–67%) of bacteria was found to be intracellular, with the remaining one third of bacteria extracellular [27]. In a subsequent study in 167 patients with blood or bone marrow culture confirmed typhoid fever, the median extracellular count of *S.* Typhi in the bone marrow aspirate was 2.5 CFU/mL (interquartile range [IQR], 0–10) and the intracellular count was 10.5 CFU/mL (IQR, 2–42) [28].

These observations suggest that antimicrobials used to treat enteric fever should target both intracellular and extracellular bacteria. Azithromycin reaches very high intracellular concentrations but low extracellular concentrations [29]. Several RCTs with azithromycin have demonstrated a slow microbiological clearance, indicated by positive blood culture during treatment. It is possible that this occurs because the low extracellular plasma levels do not clear the extracellular bacteria. Cefixime is predominantly active in the extracellular compartment although in vitro evidence indicates some intracellular activity [30]. The relative lack of intracellular cefixime activity may be the reason for the variable treatment results in typhoid. Similar considerations may also apply to other beta-lactam antimicrobials such as amoxycillin, ceftriaxone, and meropenem.

It is possible that a combination of both azithromycin, active mainly intracellularly, and cefixime, active mainly extracellularly, will be a better option for the treatment of enteric fever.

There is some clinical evidence supporting this combination. The clinical response to treatment in 37 Israeli travelers returning from Nepal with paratyphoid was significantly better when azithromycin was combined with ceftriaxone in comparison to ceftriaxone alone with the fever clearance times reduced from 6 to 3 days [31]. In an RCT of 105 adults with confirmed typhoid fever in Nepal, a combination of azithromycin and cefixime for outpatients and azithromycin and ceftriaxone for inpatients was superior to azithromycin alone with shorter fever clearance times [32]. Resistance to ceftriaxone was found in 1 (1%) of 105 isolates in this study and none were resistant to azithromycin.

This combination (azithromycin and ceftriaxone) should still be efficacious if the infecting pathogen was resistant to one of the drugs. It is possible that the combination may also prevent the emergence of resistance, which is a justification for combination chemotherapy in infections such as malaria, tuberculosis, and human immunodeficiency virus. If it could delay or prevent the emergence of this resistance that would have an important public health benefit. Because the use of drug combinations would lead to increased costs and potential side effects, it is critically important to establish whether there is a measurable clinical benefit of using these combinations. This potential effect is particularly important because fixed-dose combination of these antimicrobials is already being used in India [33].

This drug combination is the rationale for the ACT-SA randomized controlled trial currently in progress in South Asia [34]. The study is a phase IV, international multicenter, multicountry, comparative participant- and observer-blind, 1:1 randomized clinical trial. Patients with suspected uncomplicated enteric fever will be randomized to 1 of the 2 interventions. The first intervention is oral azithromycin and oral cefixime both given for 7 days. In the comparison arm, oral azithromycin will be given with a cefixime-matched placebo for 7 days. Participants with evidence of cefixime resistance will be included in the study. The study aims to recruit 1500 patients across sites in Bangladesh, Nepal, and Pakistan and will assess whether outcomes are better with the combination after 1 week of treatment and at 1- and 3-month follow-up. Trial completion is expected toward the end of 2024.

The study attempts to address some of the criticisms of previous RCTs in enteric fever. The trial is being conducted on outpatients, the cefixime component is double blind, and the trial is multisite and multicountry, with an adequate number of participants, and the results will be analyzed by “intention to treat” as well as culture positivity.

## ARE THERE OTHER ANTIMICROBIALS WE CAN USE FOR TREATMENT?

There is a need to consider alternative antimicrobial agents for treating enteric fever. In vitro studies have shown that other antimicrobials have activity against circulating organisms such as ertapenem, piperacillin-tazobactam, ceftazidime avibactam, and ceftolozane-tazobactam, but there is little clinical experience reported for these agents [35, 36]. A limitation of these options is the need for parenteral treatment and their expense. Oral options are required for this largely outpatient disease.

The oral carbapenem tebipenem pivoxil has been studied for in vitro activity against current enteric fever isolates [37]. The minimal inhibitory concentration (MIC) of tebipenem against 100 clinical isolates from Nepal and Pakistan including XDR and non-XDR *S.* Typhi and *S.* Paratyphi A was consistently ≤0.62 mg/L (IQR, 0.12–0.25 mg/L). Most *S.* Typhi from both countries had lower MIC values (median 0.12 mg/L and 0.039 mg/L, respectively) compared with Nepali *S.* Paratyphi A (non-XDR) (median = 0.31 mg/L). These data suggest that the drug is likely to work in patients infected with enteric fever with XDR and non-XDR isolates. In time-kill studies, 2 representative isolates were killed within 8–24 hours at a concentration of 2–4× MIC. In addition, tebipenem demonstrated synergy with azithromycin with efficient bacterial killing.

Tebipenem is licensed for pediatric respiratory infections in Japan and has a good safety record [38]. Whether it is clinically effective for treating in enteric fever requires clinical studies. There might be concerns about deploying an oral carbapenem in areas where resistance to existing carbapenems is a significant clinical problem in other Enterobacteriaceae and much antimicrobial prescribing is unregulated.

## WHAT ABOUT TREATMENT FOR CHRONIC CARRIAGE?

*Salmonella* Typhi and *S.* Paratyphi A may be shed in the feces (1) just before and during the acute illness and (2) during the few weeks of early convalescence. Occasionally, fecal shedding continues for prolonged periods when the person is labeled a “chronic carrier” (defined as excretion of the bacterium in the feces for more than 1 year). Chronic carriers may intermittently shed large numbers of bacteria in the feces and be a source of infection to others in the community [39]. The detection and eradication of chronic fecal carriage may become important if the rollout of vaccination leads to a decline in enteric fever incidence.

There is a lack of clear evidence to guide the treatment of chronic carriage. A recent systematic review of studies of the antimicrobial treatment of chronic carriage identified 8 studies but only 1 RCT [40]. The FQs have been shown to be effective in eradicating chronic carriage after a 28-day course. The only double-blinded RCT performed showed an eradication rate of 92% in those given a 28-day course of norfloxacin compared with 11% in those given placebo. Patients with and without gallstones were included in this study, and eradication rates were high in both groups (87% vs 100%). Six studies have evaluated ampicillin or amoxycillin with cure rates of approximately 70% after a 4- to 6-week course. High doses of intravenous amoxicillin may be more effective than oral administration, and cure rates are generally higher in those without gallstones. It is notable that all such studies were conducted between 1966 and 1988 before the emergence of widespread MDR and FQ resistance. There is no clinical outcome data to establish whether ciprofloxacin is effective in eradicating chronic carriage in isolates with low-level ciprofloxacin resistance. Azithromycin may be alternative for chronic carriage, but there is no published evidence. Cholecystectomy is a further option in recalcitrant cases but is not 100% effective. The benefit of surgery should be balanced with the risk of surgical complications, and there should be additional indications for the operation [1, 40]. Clinical trials in this area would help guide management.

## CONCLUSIONS

The case management of suspected enteric fever in outpatient departments and hospital wards is a challenge clinicians face throughout endemic areas. Inadequate diagnostics mean that most treatments are given empirically. Antimicrobial resistance to commonly used agents is widespread. There are limitations in the randomized controlled trials on which the current treatment evidence is based. Future studies should explore the clinical effectiveness of alternative antimicrobials and the value of antimicrobial combinations in acute enteric fever and chronic fecal carriage.
